# Associations Among Multimorbid Conditions in Hospitalized Middle-aged and Older Adults in China: Statistical Analysis of Medical Records

**DOI:** 10.2196/38182

**Published:** 2022-11-24

**Authors:** Yan Zhang, Chao Chen, Lingfeng Huang, Gang Liu, Tingyu Lian, Mingjuan Yin, Zhiguang Zhao, Jian Xu, Ruoling Chen, Yingbin Fu, Dongmei Liang, Jinmei Zeng, Jindong Ni

**Affiliations:** 1 Precision Key Laboratory of Public Health School of Public Health Guangdong Medical University Dongguan China; 2 Department of Elderly Health Management Shenzhen Center for Chronic Disease Control Shenzhen China; 3 Institute of Public Health and Wellness Guangdong Medical University Dongguan China; 4 Department of Primary Public Health Promotion Shenzhen Center for Disease Control and Prevention Shenzhen China; 5 Administration Office Shenzhen Center for Chronic Disease Control Shenzhen China; 6 Faculty of Education, Health and Wellbeing University of Wolverhampton Wolverhampton United Kingdom

**Keywords:** multimorbidity, chronic conditions, aging, association rule mining, decision tree analysis

## Abstract

**Background:**

Multimorbidity has become a new challenge for medical systems and public health policy. Understanding the patterns of and associations among multimorbid conditions should be given priority. It may assist with the early detection of multimorbidity and thus improve quality of life in older adults.

**Objective:**

This study aims to comprehensively analyze and compare associations among multimorbid conditions by age and sex in a large number of middle-aged and older Chinese adults.

**Methods:**

Data from the home pages of inpatient medical records in the Shenzhen National Health Information Platform were evaluated. From January 1, 2017, to December 31, 2018, inpatients aged 50 years and older who had been diagnosed with at least one of 40 conditions were included in this study. Their demographic characteristics (age and sex) and inpatient diagnoses were extracted. Association rule mining, Chi-square tests, and decision tree analyses were combined to identify associations between multiple chronic conditions.

**Results:**

In total, 306,264 hospitalized cases with available information on related chronic conditions were included in this study. The prevalence of multimorbidity in the overall population was 76.46%. The combined results of the 3 analyses showed that, in patients aged 50 years to 64 years, lipoprotein metabolism disorder tended to be comorbid with multiple chronic conditions. Gout and lipoprotein metabolism disorder had the strongest association. Among patients aged 65 years or older, there were strong associations between cerebrovascular disease, heart disease, lipoprotein metabolism disorder, and peripheral vascular disease. The strongest associations were observed between senile cataract and glaucoma in men and women. In particular, the association between osteoporosis and malignant tumor was only observed in middle-aged and older men, while the association between anemia and chronic kidney disease was only observed in older women.

**Conclusions:**

Multimorbidity was prevalent among middle-aged and older Chinese individuals. The results of this comprehensive analysis of 4 age-sex subgroups suggested that associations between particular conditions within the sex and age groups occurred more frequently than expected by random chance. This provides evidence for further research on disease clusters and for health care providers to develop different strategies based on age and sex to improve the early identification and treatment of multimorbidity.

## Introduction

### Background

China is the world’s most populous country and has the largest aging population. The population aged 65 years and older has markedly increased in recent years, and there were approximately 190 million people aged 65 years and older in China in 2020 [1]. With such a large aging population, chronic conditions are a major contributor to health burden, inequalities in health outcomes, and economic burden in China [2]. Multimorbidity (defined as 2 or more coexisting chronic conditions) has become a new challenge for medical systems and public health policy [3-5]. Multimorbidity is often associated with functional limitations, reduced quality of life, higher mortality, higher rates of adverse drug events, and frequent use of health services [6,7]. Despite the growing number of studies suggesting that multimorbidity is normal for older adults, the majority of health care systems and public health policies is focused on the treatment of individual diseases rather than a complex network of diseases [3]. The incidence of multimorbidity is latent, and the progression is slow [8]. If early detection and diagnosis are not efficient and timely, this not only will delay treatment and prognosis and affect the development of the disease but also may lead to premature death [9]. Therefore, understanding the patterns and associations among multimorbid conditions should be given priority, which may assist the early diagnosis of multimorbidity and thus improve quality of life of older adults [10].

An increasing number of studies have reported on the frequent combinations of diseases and described the patterns of multimorbidity. These studies used various methods, such as generating all possible combinations of chronic diseases, estimating observed-to-expected ratios or relative risk among the most common combination of 2 or 3 chronic conditions [11], cluster analysis [12,13], latent class analysis [14,15], factor analyses [16,17], and network analysis [4,18]. These methods are similar and investigate combinations of conditions but do not elucidate associations and the prioritization of associations between individual conditions. Furthermore, these disease combinations are mainly based on a single algorithm and lack further methods to verify their stability.

Association rule mining (ARM) is now being used to explore associations between frequent diseases [6]. ARM, a data mining technique used extensively in health care, attempts to identify and predict rules by extracting simple structures from a set of items in a database [19]. However, extrapolation of the association results based on existing samples and the priority of the associated condition of the target conditions are not taken into account in traditional ARM. With the addition of the Chi-square test and decision tree analysis, these disadvantages can be avoided. The Chi-square test is a statistical method based on the difference in rate distribution, which can be used to test the statistical significance of the associations between the antecedent conditions and the consequent conditions in the association results, in order to extrapolate the sample results to the population situation. Decision tree analysis, a powerful statistical tool, has been successfully applied to recursively split independent variables into groups to predict an outcome [20,21]. In previous studies, it was also utilized to explore associated factors with survival in breast cancer patients [22], examine the interaction of shared variables to predict survival in patients with newly diagnosed malignant pleural mesothelioma [23], and investigate the prognostic importance of each factor for overall survival [24]. Unlike common methods, decision tree analysis can be used to classify factors to determine their importance to the target variables and decide which factor has the strongest association with the dependent variable at each point in the tree structure [25]. The combination of the 3 methods can obviously strengthen the evidence of the association between conditions, which enables accurate clinical decision support in practice. For more details on comparisons with currently used methods, please refer to Multimedia Appendix 1.

In addition, most studies on multimorbidity in China were conducted in community-dwelling populations, and self-reported questionnaires were used to define chronic diseases, which may have been affected by recall and reporting bias [11,26]. Hospital medical records describe the occurrence, development, diagnosis, and treatment of patients, and more objective clinical diagnoses are used to define multimorbidity. Obtaining the medical records of hospitalized patients to study multimorbidity could avoid recall or reporting bias. Furthermore, although multimorbidity is strongly associated with sociodemographic factors, few studies have focused on multimorbidity associations by age and sex.

### Objectives

To better understand the multimorbidity patterns in middle-aged and older people, this study used the novel method of combining ARM with a traditional statistical significance test and decision tree analysis to examine and compare associations among multimorbid conditions by age and sex in a large number of middle-aged and older Chinese adults using the home pages of inpatient medical records in Shenzhen, China. It was hoped that the results would provide possible potential trajectories between multimorbid conditions and improve population-specific approaches to early detection and management of multimorbidity.

## Methods

### Data Source

This study used data from the home pages of inpatient medical records in the Shenzhen National Health Information Platform, a data center that collects medical information on cases from all medical institutions in Shenzhen. The home pages of inpatient medical records, including information on hospitalized patients’ demographic characteristics (age and sex), inpatient diagnoses, International Classification of Diseases version 10 (ICD-10) codes, and personal identifiers, were removed. All clinical visits by patients were linked to their unique encrypted identification number.

### Measurement of Multimorbidity and Study Population

In this study, the following 40 chronic conditions were selected based on the most frequently mentioned diseases in multimorbidity by previous studies that were considered to significantly impact long-term treatment and quality of life among middle-aged and older Chinese individuals [7,27]: hypertension (HT), diabetes mellitus (DM), lipoprotein metabolism disorder (LMD), chronic gastritis, chronic obstructive pulmonary disease, cerebrovascular disease (CBD), chronic kidney disease (CKD), spleen disease, peripheral vascular disease (PVD), varicose veins, schizophrenia, malignant tumor (MT), dementia, Alzheimer disease, bronchiectasis, glaucoma, senile cataract (SC), asthma, chronic nasopharyngitis, chronic viral hepatitis, thyroid disorders, hearing loss, dermatitis and eczema, anemia, migraine, chronic liver disease (CLD), depression, epilepsy, anxiety, Parkinson disease, sleep disorder, heart disease (HD), chronic gastric ulcer, gout, osteoporosis, transient cerebral ischemia, arthropathy, spondylosis, and dizziness/vertigo. Conditions were identified if they had been documented using inpatient ICD-10 codes in an individual’s medical records. Multimedia Appendix 2 lists all chronic conditions included and their corresponding ICD-10 codes. For this study, multimorbidity was defined as having 2 or more concurrent chronic conditions.

In this study, patient inclusion criteria included (1) diagnoses with at least one of the aforementioned 40 conditions in all inpatient records from January 1, 2017, to December 31, 2018, and (2) aged 50 years or older on earlier records. Middle-aged patients with multimorbidity represent a large group, and the prevalence of multimorbidity ranges from 45% to 72% among middle-aged and older people older than 50 years [28]. Exclusion criteria were that none of the conditions were diagnosed in any inpatient records during the study period. A total of 306,264 patients were included.

### Statistical Analyses

#### Descriptive Statistics

First, descriptive statistics were used in the study population, including number, proportion (%), median, and IQR of age for sex (female and male). The top 10 prevalent chronic conditions with the largest composition ratio, including the average number (mean [SD]) of coexisting conditions, were evaluated. Furthermore, age was categorized into 2 subgroups (50-64 years and ≥65 years) and cross-combined with sex into 4 age-sex subgroups. The number and proportion were used to describe the distribution of patients with or without multimorbidity, and the Chi-square test was performed to compare differences in the characteristics of patients with and without multimorbidity.

#### Association Rule Mining Based on Subgroups

To identify the associations between conditions by age and sex, 4 age-sex–based subgroup analyses were then performed. ARM was applied to determine common multimorbidity patterns that met a minimum requirement of measurement indicators. Association rules were relationships between sets of conditions from “antecedent” to “consequent” [29]. We used 3 common measurement indicators: (1) support (how frequently the condition combinations appear in the data set), (2) confidence (how frequently the consequent conditions occur, conditional on the antecedent conditions), and (3) lift (the ratio of the observed support to that expected if antecedent and consequent were independent) [30]. Lift was considered the main measure of significance in ARM. A lift of “1” means that the probabilities of occurrence of the antecedent and consequent are independent of each other. Hence, a higher lift indicates a higher chance of co-occurrence of the consequent with the antecedent and a more significant association [31]. Setting a higher threshold value would reduce the number of rules that might result in missing essential rules with low frequencies, and setting a lower threshold value could result in a large number of rules that might hinder the management from summarizing rules [29]. Thus, many rounds of testing and evaluation were carried out before defining final thresholds to mine reasonable rules and to ensure the robustness of the model performance. Considering the vast number of disease types in the data set, the rules satisfying support >1%, confidence >50%, and lift >1 were selected. All association rules were sorted by lifts, and the top 10 association rules with larger lifts in 4 subgroups were described.

#### Chi-square Tests

To evaluate the statistical significance of the aforementioned association rules, Chi-square tests were applied. Odds ratios (ORs) and 95% CIs between antecedent conditions and consequent conditions in the association rules of the 4 age-sex subcategories are shown.

#### Decision Tree Analysis

Furthermore, decision tree analysis was conducted to examine the conditions associated with the main consequent conditions in the association rules. Decision tree analysis examines the relationship between influencing factors and target variables [32]. The decision tree process is a nonparametric method that creates a tree-based classification model [33]. A decision tree contains 3 main parts: decision nodes, branches, and leaves. The internal variables of the model represent a tree structure in which a decision is made in each branch according to the data features [25]. The tree starts with a node and extends to the leaf. The risky paths are identified and shown in nodes [34]. In this study, we used decision tree analysis to determine the relationship between the conditions and main consequent conditions in rule results. Thus, the consequent conditions in rule results were used as target variables, while the remaining conditions were used as the independent variables. Splitting criteria provides a rate for each predictor variable. Variables that have the best rate of splitting criteria are selected to remain in the model [25], which have a greater impact on the target variables, and in this study, various conditions were screened based on this feature. In the decision tree, the first variable or root node is the most important factor, and other variables can be classified in order of importance [35]. The decision trees were drawn to show the associated conditions with main consequent conditions in association rules.

**Figure 1:**
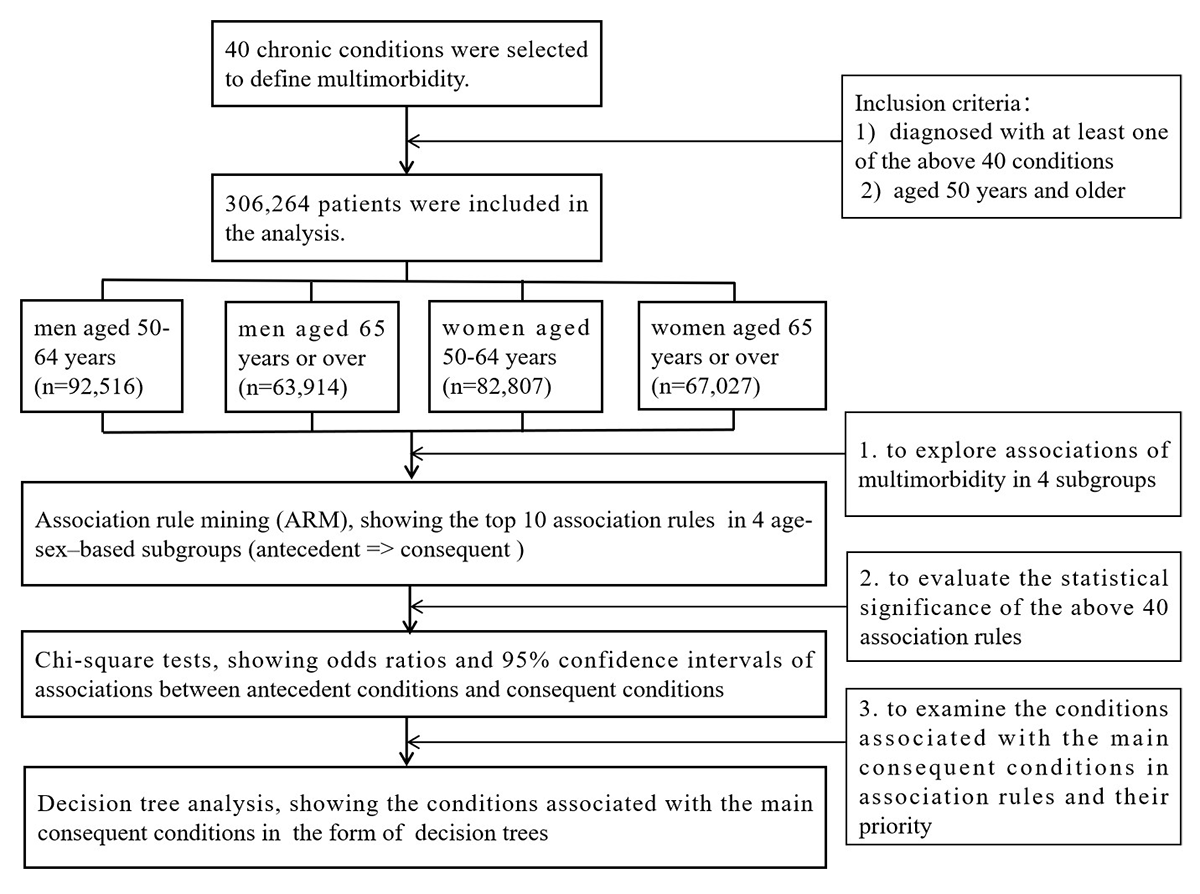
The flowchart of the main research steps.

The flowchart of the analyses is shown in Figure 1. All descriptive analyses and Chi-square tests were performed using SPSS version 25.0 (IBM Corp, Armonk, NY), with a .05 level of significance. ARM and decision tree analysis were carried out using R 3.4.0 (The R Foundation for Statistics and Mathematics, Vienna, Austria) with the arules package and the tree package. To make the results more intuitive, GraphPad Prism version 8.0 (GraphPad Software, San Diego, CA) was used to show the ORs and 95% CIs, and PowerPoint software 2021 version (Microsoft Corp, Redmond, WA) was used to draw decision trees.

### Ethics Approval

This study was approved by the Institutional Review Ethics Committee of the Affiliated Hospital of Guangdong Medical University (YJYS202008). Informed consent was not required from participants as all data provided were deidentified.

## Results

### Characteristics of the Participants

In total, 306,264 hospitalized cases with available information on related chronic conditions were included in this study. The median age of the study population was 62 (IQR 55-71) years. There were more men than women, with men accounting for 51.08% (156,430/306,264) of the sample. The median age of the male and female participants was 62 (IQR 54-71) years and 63 (IQR 56-72) years, respectively (Table 1).

**Table 1:** Characteristics of the study population (N=306,264).

Characteristics	All respondents	Men	Women
Number of people, n (%)	306,264 (100)	156,430 (51.08)	149,834 (48.92)
Age (years), median (IQR)	62 (55-71)	62 (54-71)	63 (56-72)

### Characteristics of the Chronic Conditions

As shown in Table 2, 44.72% (136,972/306,264) of the study population had HT, which was the most prevalent condition. This was followed by HD (74,535/306,264, 24.34%), DM (70,917/306,264, 23.16%), CBD (68,151/306,264, 22.25%), LMD (65,385/306,264, 21.35%), CKD (63,470/306,264, 20.72%), CLD (61,829/306,264, 20.19%), PVD (51,311/306,264, 16.75%), spondylosis (42,982/306,264, 14.03%), and gout (33,984/306,264, 11.10%). Patients with these chronic conditions had an average multimorbidity burden of ≥4 chronic conditions per patient.

**Table 2:** Top 10 conditions with the largest composition ratio in all cases (N=306,264).

Rank	Chronic conditions	Presence in all participants, n (%)	Number of co-occurring conditions, mean (SD)
1	Hypertension	136,972 (44.72)	4.79 (0.76)
2	Heart disease	74,535 (24.34)	4.97 (0.27)
3	Diabetes mellitus	70,917 (23.16)	4.84 (0.67)
4	Cerebrovascular disease	68,151 (22.25)	4.89 (0.53)
5	Lipoprotein metabolism disorder	65,385 (21.35)	4.91 (0.49)
6	Chronic kidney disease	63,470 (20.72)	4.81 (0.77)
7	Chronic liver disease	61,829 (20.19)	4.87 (0.59)
8	Peripheral vascular disease	51,311 (16.75)	4.95 (0.38)
9	Spondylosis	42,982 (14.03)	4.94 (0.39)
10	Gout	33,984 (11.10)	4.97 (0.29)

### Differences in the Characteristics of Patients With and Without Multimorbidity

Of the 306,264 patients included, over 50% (175,323/306,264, 57.25%) were between 50 years and 64 years old (Table 3). The prevalence of multimorbidity in the overall population was 76.46% (234,156/306,264), with a higher prevalence in patients aged 65 years or older (108,937/306,264, 83.20%) than in those aged 50 years to 64 years (125,219/306,264, 71.42%). There were statistically significant sex differences in the prevalence of multimorbidity in the overall population, and patients aged 50 years to 64 years showed a higher prevalence in men than in women.

**Table 3:** Differences in the characteristics of patients with and without multimorbidity (N=306,264).

Age groups			Multimorbidity, n (%)		No multimorbidity, n (%)		*P* value
≥**50 and ≤64 years**							
	Men	67,665 (73.14)		24,851 (26.86)		<.001	
	Women	57,554 (69.50)		25,253 (30.50)			
	Total	125,219 (71.42)		50,104 (28.58)		—^a^	
≥**65 years**							
	Men	53,305 (83.40)		10,609 (16.60)		.052	
	Women	55,632 (83.00)		11,395 (17.00)			
	Total	108,937 (83.20)		22,004 (16.80)		—	
**Overall sample**							
	Men	120,970 (77.33)		35,460 (22.67)		<.001	
	Women	113,186 (75.54)		36,648 (24.46)			
	Total	234,156 (76.46)		72,108 (23.54)		—	

^a^Not applicable.

### Association Rules and Statistical Analysis Results

The top 10 association rules in 4 age-sex–based subgroups according to lifts are shown in Multimedia Appendix 3. Among men and women aged 50 years to 64 years, LMD tended to be comorbid with DM, CLD, gout, HT, and PVD, which occurred in 7 association rules in men and in 10 rules in women. In addition, the combination of osteoporosis and MT was observed to have the strongest association in men, with a lift of 6.60, whereas this combination was not found in women. For patients aged 65 years or older, PVD tended to be present in combination with HT, LMD, CBD, and HD, which occurred in 5 association rules in men and 4 in women among the top 10 rules, indicating that these antecedent combinations positively correlated with the occurrence of PVD. Furthermore, the strongest associations were observed between SC and glaucoma in men (lift=6.65) and in women (lift=4.93). In particular, the 4 association rules including osteoporosis and MT were only observed in men, and their lifts were all greater than 4, while the associations between anemia, gout, and CKD (lift=3.00) were only observed in women. 

**Figure 2:**
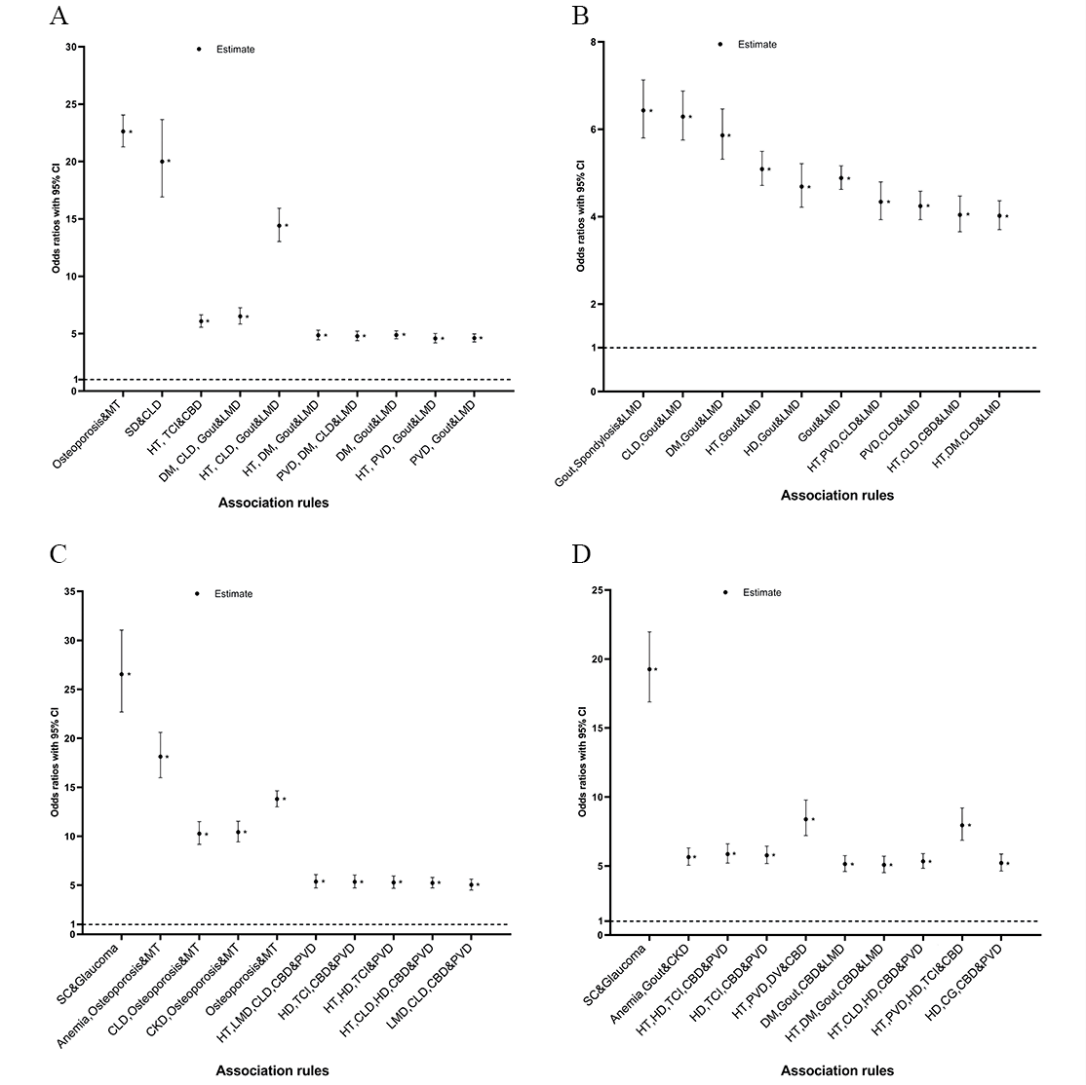
Point estimates of the odds ratios and 95% CIs (1.96 SE) of the associations between antecedent conditions and consequent conditions in the association rules of 4 age-sex subgroups in (A) men aged 50-64 years, (B) women aged 50-64 years, (C) men aged 65 years or older, and (D) women aged 65 years or older. The * indicate significant findings. CBD: cerebrovascular disease; CG: chronic gastritis; CKD: chronic kidney disease; CLD: chronic liver disease; DM: diabetes mellitus; DV: dizziness/vertigo; HD: heart disease; HT: hypertension; LMD: lipoprotein metabolism disorder; MT: malignant tumor; PVD: peripheral vascular disease; SC: senile cataract; SD: spleen disease; TCI: transient cerebral ischemia.

Statistical analysis (Chi-square tests) of the association rules in 4 age-sex–based subgroups was carried out, and the results are shown in Figure 2 and Multimedia Appendix 4. For all 40 rules, the ORs of the associations between antecedent conditions and consequent conditions were greater than “1,” and the 95% CIs did not include “1,” indicating that the latter conditions were more likely to be positive when the combinations of antecedent conditions were positive than negative.

### Decision Tree Analysis of the Main Association Rules

**Figure 3:**
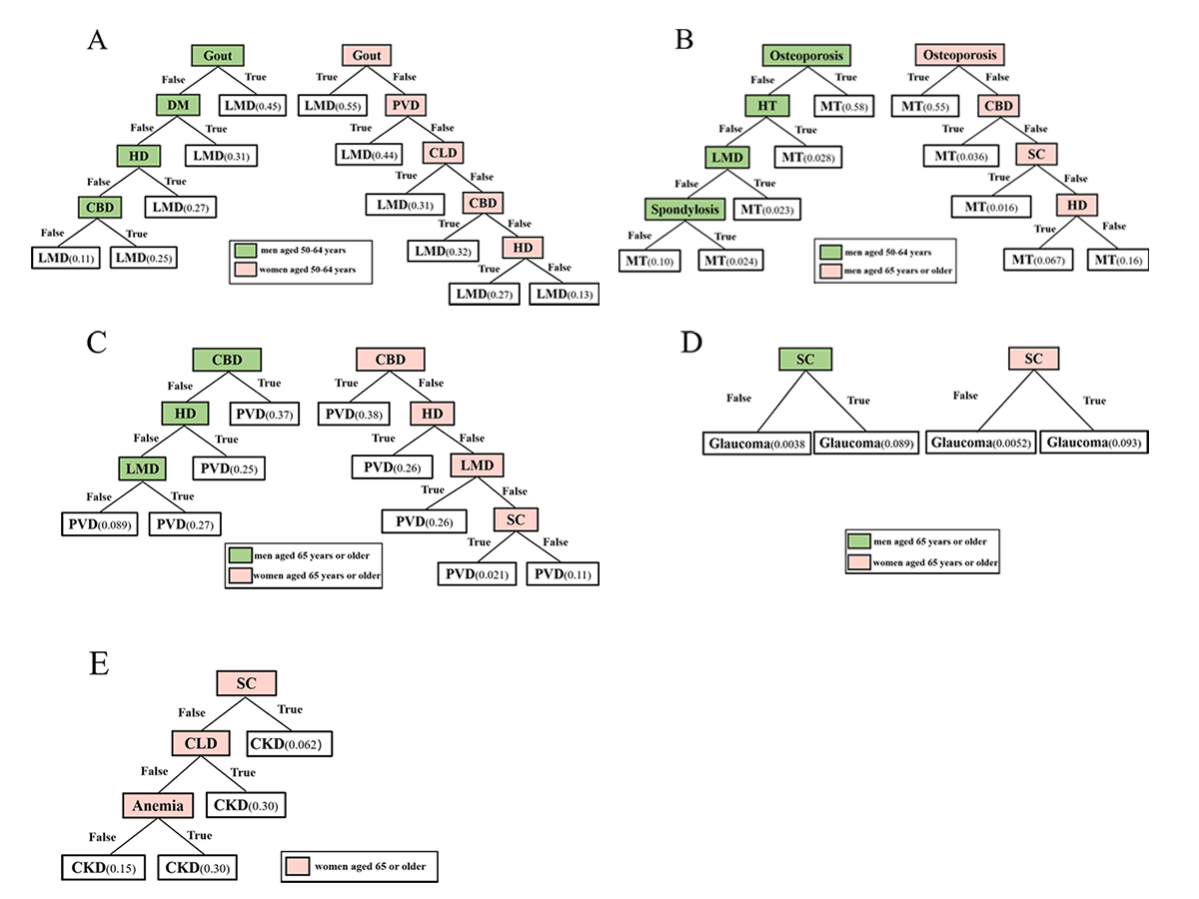
Decision trees with main consequent conditions as the target variables (other conditions divided into 2 subcategories: positive = “True”; negative = “False) in the association rules of different age-sex subgroups: (A) lipoprotein metabolism disorder (LMD) as the target variable in men and women aged 50-64 years, (B) malignant tumor (MT) as the target variable in men aged 50-64 years and 65 years or older, (C) peripheral vascular disease (PVD) as the target variable in men and women aged 65 years or older, (D) glaucoma as the target variable in men and women aged 65 years or older, (E) chronic kidney disease (CKD) as the target variable in women aged 65 years or older. All decimal values represent the proportion of the target conditions that were positive when the associated conditions were in the corresponding subgroup. CBD: cerebrovascular disease; CLD: chronic liver disease; DM: diabetes mellitus; HD: heart disease; HT: hypertension; SC: senile cataract.

Decision tree analysis was used to examine the associated comorbidities of the main consequent conditions in the rule results. The main decision trees are shown in Figure 3. Figure 3A shows that, in patients aged 50 years to 64 years, the decision tree with LMD as the dependent variable included nodes of gout, DM, HD, and CBD in men, and gout was at the top of the tree, indicating that 45.05% (5830/12,940) of patients with gout had LMD. More importantly, gout, CBD, and HD remained in the LMD decision tree for women. Gout was still at the top of the tree, and 55.37% (3270/5920) of patients with gout had LMD. Figure 3B shows that, in men aged 50 years to 64 years and 65 years or older, osteoporosis remained at the top of the decision tree of MT in men, indicating that more than 50% of patients with osteoporosis had a comorbidity of MT. Among patients aged 65 years or older, condition nodes reserved in the decision tree of PVD included CBD, HD, and LMD in men and CBD, HD, LMD, and SC in women (Figure 3C). SC was the only node in the glaucoma decision tree in both sexes (Figure 3D). Furthermore, in women, SC, CLD, and anemia were observed in the decision tree of CKD (Figure 3E).

## Discussion

### Principal Findings

Understanding multimorbidity associations is an important public health priority for clinicians, academics, and funders alike [9]. This study was conducted to comprehensively evaluate the associations among multimorbid conditions based on the electronic hospitalized medical record home pages of a large sample of middle-aged and older Chinese people. To the best of our knowledge, this study is the first to evaluate multimorbidity associations using a comprehensive analysis with ARM, Chi-square tests, and decision tree analysis. Our analysis process not only revealed associations between particular conditions within different age-sex subgroups but also examined the importance of these associated chronic conditions for certain target conditions.

In this study of more 300,000 cases, more than 76% of patients were found to have 2 or more chronic conditions in the comprehensive list of 40 chronic conditions examined. The results showed that multiple conditions including HT, HD, DM, CBD, and LMD were the most common among hospitalized middle-aged and older Chinese adults, and they co-occurred with more than 3 other conditions. This is similar to the findings reported in previous studies [4,7]. The prevalence of multimorbidity varied across the 2 age groups (50-64 years old and ≥65 years old) and both sex groups, reflecting the strong associations between multimorbidity and both age and sex [6]. Therefore, our subsequent analysis was based on specific age-sex subgroups to identify and compare the associations among multimorbid conditions within age and sex.

Association rules can reflect the interdependence and relevance between one condition and others. In our study, the ranked lift of most association rules indicated that LMD was the dominant condition among men and women aged 50 years to 64 years and was directly and indirectly associated with multiple conditions, including DM, CLD, gout, HT, and PVD or combinations of these conditions, which was also confirmed by statistical analysis. The potential mechanisms might include increased systemic inflammatory mediators and some adverse effects, such as physical inactivity, which are also risk factors for associated conditions [7]. Furthermore, in both men and women, gout appeared at the top of the decision tree with LMD as the dependent variable, which proved that the strong association between gout and LMD was not coincidental. Our findings are consistent with those from previous studies. In a review, the author concluded that complex interconnections between gout and metabolic syndromes including LMD existed, showing that gout may play an important role in the manifestation of metabolic syndromes [36]. Therefore, proper management of one disease may have implications for early detection and prevention of another.

Among patients aged 65 years or older, ARM, statistical analysis, and decision tree analysis consistently found that PVD was closely interlinked with CBD, HD, and LMD. It was previously reported that these diseases share risk, pathophysiological, and prognostic features and their coexistence would cause a cumulative burden [27]. People with PVD are at significantly higher risk of myocardial infarction and stroke than the general population [37]. Although PVD can lead to adverse health outcomes, it has received little attention [38]. As an important comorbidity, PVD needs to be emphasized, and patients diagnosed with associated conditions should be targeted for PVD screening. Similarly, a significant association between SC and glaucoma was confirmed by all 3 methods, indicating that the probability of glaucoma was higher than the probability of other conditions when SC was present. This finding was consistent with that in a study based on large medical claims data among a Chinese population of 2 million [7]. The incidence of glaucoma and comorbid SC will increase with age, and measurements targeting those shared specific factors may benefit 2 or more related diseases [39].

Men aged 50 years to 64 years or 65 years or older reported a high prevalence of MT, with a high probability of co-occurrence with osteoporosis in the association rules and statistical analysis, which was consistent with the results of the decision tree analysis. Osteoporosis was found to be the condition most related with MT. Certain biological links have indeed been found between osteoporosis and MT, including the presence of important cytokines, hormones, and oxidative stress [40]. However, the sex difference between the 2 conditions in our research was inconsistent with some previous studies, which showed that osteoporosis and some types of MT, including breast cancer, thyroid cancer, and colorectal cancer, were more closely linked in women than in men [40-43]. This may be affected by factors such as MT type and age of the population, which requires further investigation in cancer subgroups. However, recognizing the existence of this association may help to guide the early screening of MT in Chinese middle-aged and older men with osteoporosis, especially the type with a high incidence in men.

The strong association between anemia, gout, and CKD was only detected in women aged 65 years or older by ARM and statistical analysis. The lift of 3.00 indicated that these conditions were 3 times as likely to occur simultaneously as they were alone. In the decision tree analysis, SC, CLD, and anemia were observed to be CKD-associated conditions. The common results of these 3 methods seemed to imply that there was a special association between anemia and CKD in this subgroup. Anemia is a common complication and contributes to increased morbidity and mortality in CKD patients, which has been demonstrated previously [44,45]. A systematic review concluded that excess was a main contributor to the disordered iron homeostasis and anemia of CKD by impairing dietary iron absorption and iron mobilization from body stores [46]. Furthermore, possible explanations for this relationship only found in older women included shared risk factors of 2 conditions, such as aging and female sex [47,48]. Therefore, for older women, active improvement of anemia may be of great significance in preventing and delaying the development of CKD.

The main strength of this study is that a novel method was used, that is, the combination of ARM with a traditional statistical significance test and decision tree analysis, to examine the associations of multimorbidity. In particular, this was the first time that decision tree analysis was used in a multimorbidity study. Second, the disease diagnoses that defined multimorbidity in our analysis were based on a large sample of inpatient medical records, which avoided recall or reporting bias. Finally, our association analysis was based on age-sex subgroups, avoiding the confounding effects of age and sex. The present findings indicated that combinations of particular conditions within sex and age groups occur more frequently than expected by random chance. This provides evidence for further research on the potential mechanisms and risk factors for specific combinations and to encourage health care providers to develop population-specific approaches for early detection and management of multimorbidity according to sex and age.

### Limitations

Several limitations of our study must be acknowledged. First, our sample consisted of hospitalized cases, and mild and early cases may not have been included. In view of the fact that the research on multimorbidity in China is still at an early stage, our findings based on more severe cases may provide ideas for research on the early prevention of combinations of specific conditions. Second, we could not draw conclusions about causality effects between multiple conditions due to the cross-sectional design of the study. Finally, patients’ socioeconomic status, family history, and lifestyle factors were not incorporated into the model in this analysis due to data availability, and the data set anonymized participants to avoid possible misuse; therefore, some potential confounding factors were not taken into consideration. However, given the advantages of our large sample size, the findings do provide support and a new perspective for future longitudinal or experimental studies to identify potential mechanisms and risk factors for specific combinations.

### Conclusions

Multimorbidity was prevalent among middle-aged and older Chinese individuals. The results of this comprehensive analysis of 4 age-sex subgroups suggested that associations among particular conditions within sex and age groups occurred more frequently than expected by random chance. This provides evidence for further research on disease clusters and for health care providers to develop different strategies, according to age and sex, to improve the early identification and treatment of multimorbidity.

## Appendix

Multimedia Appendix 1 Comparison of methods used in multimorbidity studies.

Multimedia Appendix 2 ICD-10 numbers of 40 conditions included in the analysis.

Multimedia Appendix 3 The top 10 association rules in the order of lifts.

Multimedia Appendix 4 The results of the statistical analysis of association rules.

## Abbreviations

ARM: association rule mining
CBD: cerebrovascular disease
CKD: chronic kidney disease
CLD: chronic liver disease
DM: diabetes mellitus
HD: heart disease
HT: hypertension
ICD-10: International Classification of Diseases version 10
LMD: lipoprotein metabolism disorder
MT: malignant tumor
OR: odds ratio
PVD: peripheral vascular disease
SC: senile cataract
