# Supplementary material for: Associations Among Multimorbid Conditions in Hospitalized Middle-aged and Older Adults in China: Statistical Analysis of Medical Records
Source: JMIR Public Health Surveill. 2022 Nov 24;8(11):e38182. doi: 10.2196/38182 (PMC9732753; doi:10.2196/38182)
Supplement: Multimedia Appendix 1 [file publichealth_v8i11e38182_app1.docx]

**Comparison of methods used in multimorbidity studies**

| **Methods** | **References** | **The function or principle of the methods** | **Main features of the methods** | **Variables used in the methods** |
| --- | --- | --- | --- | --- |
| Cluster analysis | References [12, 13] | Cluster analysis obtains the patterns of multimorbidity based on dissimilarities between diseases; clusters tend to contain diagnoses that are similar to each other (in terms of Euclidean distances) and a diagnosis cannot be included in more than one cluster. | Due to the exploratory nature of cluster analysis, there was no set number of optimal clusters to generate, and different clustering algorithms may generate different numbers of clusters and constituents within the clusters, and the algorithms are not efficient to analyse large data sets, as they require a large distance matrix. Another important limitation was that each diagnosis can belong to only 1 cluster at a time. | Chronic diseases used to define multimorbidity |
| Factor analysis | References [16,17] | The factor analysis was used to identify sets of variables with a common underlying causal factor. It is based on correlations between diagnoses to identify the pattern. | Factor analysis with tetrachoric correlations led to different groupings of multimorbid conditions when different model-selection and/or rotation methods were used. | Chronic diseases used to define multimorbidity |
| Network analysis | References [4,18] | An edge or connection between two diseases is created if these are comorbid. A multimorbidity network developed from patients contains a set of nodes connected through edges. Pairwise associations between morbidities were visualised using network graphs. | If two unrelated NCDs with high prevalence, it would appear in the visualization results of network analysis. The result of network analysis was equivalent to the $sup$ in Association Rule Mining (ARM), which was just part of ARM's results. | Chronic diseases used to define multimorbidity |
| Latent class analysis | References [14,15] | Latent Class Analysis (LCA) is a statistical model in which individuals can be classified into mutually exclusive and exhaustive types, or latent classes, based on their pattern of answers on a set of (categorical) measured variables. LCA utilises maximum likelihood estimation to identify groups of cases with similar probabilities of a particular diagnosis and uses raw data as input. | Latent class analysis identifies probabilistic rather than deterministic subgroups based on responses to a set of observed variables, and assumes that the pattern is explained by unobserved categorical latent variables of K classes. | Chronic diseases used to define multimorbidity |
| Association Rule Mining (ARM) | References [6,7] | ARM is a procedure that reveals interdependence and association between different factors, and is an important technique in data mining as it can extract valuable correlated data items from large amounts of data and reflect the degree of association. ARM consists of two steps: first, it involves listing all high-frequency items in the set; second, it generates frequent association rules based on the high-frequency items. | The extrapolation of the association results based on existing sample and the priority associated variables order of the target variables are not taken into account in traditional ARM. | Chronic diseases used to define multimorbidity |
| Decision Tree Analysis | no studies of multimorbidity using this method | A decision tree analysis could provide the essential information on the characteristics that are closely associated with the outcome(s) that a researcher targets. This method can be performed on most medical data for the purpose of diagnosis and prediction. The results are displayed in a tree-like structure, which greatly facilitates the recognition and application of the results by clinicians. A decision tree contains three main parts: decision nodes, branches, and leaves. The tree starts with a node and extends to the leaf. The risky paths are identified and shown in several nodes. | The main characteristic of decision tree is the graphical display of the choices. Unlike common methods, decision tree analysis can be used to classify factors in order to determine their importance to the target variables and decide which factor has the strongest association with the dependent variable at each point in the tree structure. This advantage provides alternatives for each decision and possible outcomes and allows comparisons of different alternatives. | — |
