# Supplementary material for: Associations Among Multimorbid Conditions in Hospitalized Middle-aged and Older Adults in China: Statistical Analysis of Medical Records
Source: JMIR Public Health Surveill. 2022 Nov 24;8(11):e38182. doi: 10.2196/38182 (PMC9732753; doi:10.2196/38182)
Supplement: Multimedia Appendix 2 [file publichealth_v8i11e38182_app2.docx]

**ICD-10 numbers of 40 conditions included in the analysis**

| **Conditions** | **ICD-10** | **Conditions** | **ICD-10** |
| --- | --- | --- | --- |
| hypertension (HT) | I10-I15 | chronic viral hepatitis (CVH) | B18 |
| diabetes mellitus (DM) | E10-E14 | thyroid disorders(TD) | E02-E03、E05 |
| lipoprotein metabolism disorder (LMD) | E78 | hearing loss(HL) | H90-H91 |
| chronic gastritis(CG) | K29.3-K29.5 | Dermatitis and eczema | L20-L30 |
| chronic obstructive pulmonary disease(COPD) | J44 | Anemia | D50-D64 |
| cerebrovascular disease (CBD) | I60-I69 | Migraine | G43、G44 |
| chronic kidney disease (CKD) | N03-04、N06-N08、N11、N13 | chronic liver disease (CLD) | K70、K71.3-K71.5、K71.7、K72.1、K73-E76 |
| gallstone disease(GD) | K80 | Depression | F32-F33 |
| spleen disease(SD) | D73 | Epilepsy | G40 |
| peripheral vascular disease (PVD) | I70-I73.9 | Anxiety | F40-F41 |
| varicose veins(VV) | I83 | Parkinson’s disease (PD) | G20-G22 |
| schizophrenia(SP) | F20 | sleep disorder(SD) | F51、G47 |
| malignant tumor(MT) | C00-C97 | heart disease (HD) | I05-I09、I20-I27、I34-I37、I44-I49、I50、I51.9、 |
| Dementia | F00-F03 | chronic gastric ulcer (CGU) | K25.4-K25.9 |
| Alzheimer’s disease (AD) | G30 | Gout | E79、M10 |
| Bronchiectasis | J47 | Osteoporosis | M80-M82 |
| Glaucoma | H40-H42 | transient cerebral ischemia (TCI) | G45 |
| senile cataract (SC) | H25、H28.0-H28.2 | Srthropathy | M15-M19 |
| Asthma | J45-J46 | Spondylosis | M45-M49 |
| chronic nasopharyngitis (CN) | J31.0-31.2、J32 | Dizziness/vertigo | R42、H81.0-H82 |
