# Supplementary material for: Associations Among Multimorbid Conditions in Hospitalized Middle-aged and Older Adults in China: Statistical Analysis of Medical Records
Source: JMIR Public Health Surveill. 2022 Nov 24;8(11):e38182. doi: 10.2196/38182 (PMC9732753; doi:10.2196/38182)
Supplement: Multimedia Appendix 3 [file publichealth_v8i11e38182_app3.docx]

**The top 10 association rules in 4 age-sex–based subgroups according to lifts**

| **Rules** | | | **Lifts** | **Rules** | | | **Lifts** |
| --- | --- | --- | --- | --- | --- | --- | --- |
| **50-64 years（men）** | | | | **50-64 years（women）** | | | |
| Osteoporosis | ==> | MT | 6.60 | Gout, Spondylosis | ==> | LMD | 2.79 |
| SD | ==> | CLD | 3.57 | CLD, Gout | ==> | LMD | 2.75 |
| HT, TCI | ==> | CBD | 3.02 | DM, Gout | ==> | LMD | 2.69 |
| DM, CLD, Gout | ==> | LMD | 2.82 | HT, Gout | ==> | LMD | 2.53 |
| HT, CLD, Gout | ==> | LMD | 2.54 | HD, Gout | ==> | LMD | 2.48 |
| HT, DM, Gout | ==> | LMD | 2.51 | Gout | ==> | LMD | 2.41 |
| PVD, DM, CLD | ==> | LMD | 2.50 | HT, PVD, CLD | ==> | LMD | 2.40 |
| DM, Gout | ==> | LMD | 2.49 | PVD, CLD | ==> | LMD | 2.35 |
| HT, PVD, Gout | ==> | LMD | 2.46 | HT, CLD, CBD | ==> | LMD | 2.33 |
| PVD, Gout | ==> | LMD | 2.45 | HT, DM, CLD | ==> | LMD | 2.30 |
| **65 years or older（men）** | | | | **65 years or older（women）** | | | |
| SC | ==> | Glaucoma | 6.65 | SC | ==> | Glaucoma | 4.93 |
| Anemia, Osteoporosis | ==> | MT | 5.59 | Anemia, Gout | ==> | CKD | 3.00 |
| CLD, Osteoporosis | ==> | MT | 4.58 | HT, HD, TCI CBD | ==> | PVD | 2.74 |
| CKD, Osteoporosis | ==> | MT | 4.56 | HD, TCI, CBD | ==> | PVD | 2.71 |
| Osteoporosis | ==> | MT | 4.41 | HT, PVD, DV | ==> | CBD | 2.66 |
| HT, LMD, CLD, CBD | ==> | PVD | 2.62 | DM, Gout, CBD | ==> | LMD | 2.64 |
| HD, TCI, CBD | ==> | PVD | 2.62 | HT, DM, Gout, CBD | ==> | LMD | 2.63 |
| HT, HD, TCI | ==> | PVD | 2.60 | HT, CLD, HD, CBD | ==> | PVD | 2.63 |
| HT, CLD, HD, CBD | ==> | PVD | 2.58 | HT, PVD, HD, TCI | ==> | CBD | 2.63 |
| LMD, CLD, CBD | ==> | PVD | 2.55 | HD, CG, CBD | ==> | PVD | 2.62 |

**Note：**HT: hypertension, DM: diabetes mellitus, LMD: lipoprotein metabolism disorder, CG: chronic gastritis, CBD: cerebrovascular disease , CKD: chronic kidney disease , PVD: peripheral vascular disease, MT: malignant tumor, SC: senile cataract, CLD: chronic liver disease, HD: heart disease, TCI: transient cerebral ischemia , DV: dizziness/vertigo.
