# Supplementary material for: Associations Among Multimorbid Conditions in Hospitalized Middle-aged and Older Adults in China: Statistical Analysis of Medical Records
Source: JMIR Public Health Surveill. 2022 Nov 24;8(11):e38182. doi: 10.2196/38182 (PMC9732753; doi:10.2196/38182)
Supplement: Multimedia Appendix 4 [file publichealth_v8i11e38182_app4.docx]

**The results of the statistical analysis of association rules in 4 age-sex–based subgroups**

| **Antecedent** | | | **Consequent** | | **OR**  **(95%CI)** | ***P*** | **Antecedent** | |  | **Consequent** | | **OR**  **(95%CI)** | ***P*** |
| --- | --- | --- | --- | --- | --- | --- | --- | --- | --- | --- | --- | --- | --- |
|  |  |  | **+** | **-** |  |  |  |  |  | **+** | **-** |  |  |
|  | **50-64 years（men）** | | | | | |  | **50-64 years（women）** | | | | | |
|  | |  | MT | |  |  |  | |  | LMD | |  |  |
| Osteoporosis | | + | 3120(57.9) | 2269(42.1) | 22.62  (21.28,24.05) | <0.001 | Gout, Spondylosis | | + | 1075(65.19) | 574(34.81) | 6.43  （5.80，7.13） | <0.001 |
|  |  | - | 4992(5.73) | 82135(94.27) |  |  |  |  | - | 18304(22.55) | 62854(77.45) |  |  |
|  | |  | CLD | |  |  |  | |  | LMD | |  |  |
| SD | | + | 970(85.84) | 160(14.16) | 20.00  (16.91,23.66) | <0.001 | CLD, Gout | | + | 1409(64.34) | 781(35.66) | 6.29(5.75,6.88) | <0.001 |
|  |  | - | 21257(23.26) | 70129(76.74) |  |  |  |  | - | 17970(22.29) | 62647(77.71) |  |  |
|  | |  | CBD | |  |  |  | |  | LMD | |  |  |
| HT, TCI | | + | 1188(57.25) | 887(42.75) | 6.08  (5.56,6.64) | <0.001 | DM, Gout | | + | 1112(63.04) | 652(36.96) | 5.86(5.31,6.47) | <0.001 |
|  |  | - | 16334(18.06) | 74107(81.94) |  |  |  |  | - | 18267(22.54) | 62776(77.46) |  |  |
|  | |  | LMD | |  |  |  | |  | LMD | |  |  |
| DM, CLD, Gout | | + | 969(65.43) | 512(34.57) | 6.51  (5.85,7.26) | <0.001 | HT, Gout | | + | 1673(59.14) | 1156(40.86) | 5.09(4.71,5.50) | <0.001 |
|  |  | - | 20498(22.52) | 70537(77.48) |  |  |  |  | - | 17706(22.14) | 62272(77.86) |  |  |
|  | |  | LMD | |  |  |  | |  | LMD | |  |  |
| HT, CLD, Gout | | + | 1942(79.95) | 487(20.05) | 14.41  (13.03,15.94) | <0.001 | HD, Gout | | + | 840(58.05) | 607(41.95) | 4.69(4.22,5.21) | <0.001 |
|  |  | - | 19525(21.67) | 70562(78.33) |  |  |  |  | - | 18539(22.79) | 62821(77.21) |  |  |
|  | |  | LMD | |  |  |  | |  | LMD | |  |  |
| HT, DM, Gout | | + | 1282(58.35) | 915(41.65) | 4.87  (4.47,5.31) | <0.001 | Gout | | + | 3278(56.37) | 2537(43.63) | 4.89(4.63,5.16) | <0.001 |
|  |  | - | 20185(22.35) | 70134(77.65) |  |  |  |  | - | 16101(20.91) | 60891(79.09) |  |  |
|  | |  | LMD | |  |  |  | |  | LMD | |  |  |
| PVD, DM, CLD | | + | 1268(57.98) | 919(42.02) | 4.79  （4.39，5.22） | <0.001 | HT, PVD, CLD | | + | 908(56.12) | 710(43.88) | 4.34(3.93,4.80) | <0.001 |
|  |  | - | 20199(22.36) | 70130(77.64) |  |  |  |  | - | 18471(22.75) | 62718(77.25) |  |  |
|  | |  | LMD | |  |  |  | |  | LMD | |  |  |
| DM, Gout | | + | 1905(57.85) | 1388(42.15) | 4.89  （4.55.5.25） | <0.001 | PVD, CLD | | + | 1529(54.92) | 1255(45.08) | 4.24(3.93,4.58) | <0.001 |
|  |  | - | 19562(21.92) | 69661(78.08) |  |  |  |  | - | 17850(22.31) | 62173(77.69) |  |  |
|  | |  | LMD | |  |  |  | |  | LMD | |  |  |
| HT, PVD, Gout | | + | 1136(57.06) | 855(42.94) | 4.59  （4.19，5.02） | <0.001 | HT, CLD, CBD | | + | 854(54.43) | 715(45.57) | 4.04(3.66,4.47) | <0.001 |
|  |  | - | 20331(22.46) | 70194(77.54) |  |  |  |  | - | 18525(22.8) | 62713(77.2) |  |  |
|  | |  | LMD | |  |  |  | |  | LMD | |  |  |
| PVD, Gout | | + | 1605(56.75) | 1223(43.25) | 4.61  （4.28，4.98） | <0.001 | HT, DM, CLD | | + | 1292(53.86) | 1107(46.14) | 4.02(3.71,4.37) | <0.001 |
|  |  | - | 19862(22.15) | 69826(77.85) |  |  |  |  | - | 18087(22.49) | 62321(77.51) |  |  |
|  | **65 years or older（men）** | | | | | |  | **65 years or older（women）** | | | | | |
|  | |  | SC | |  |  |  | |  | SC | |  |  |
| Glaucoma | | + | 660(75.69) | 212(24.31) | 26.54 (22.69,31.04) | <0.001 | Glaucoma | | + | 968(76.28) | 301(23.72) | 19.27 (16.90,21.97) | <0.001 |
|  |  | - | 6619(10.5) | 56423(89.5) |  |  |  |  | - | 9407(14.31) | 56351(85.69) |  |  |
|  | |  | MT | |  |  |  | |  | CKD | |  |  |
| Anemia, Osteoporosis | | + | 824(70.13) | 351(29.87) | 18.14 (15.97,20.60) | <0.001 | Anemia, Gout | | + | 705(54.4) | 591(45.6) | 5.65  (5.05 ,6.31) | <0.001 |
|  |  | - | 7190(11.46) | 55549(88.54) |  |  |  |  | - | 11462(17.44) | 54269(82.56) |  |  |
|  | |  | MT | |  |  |  | |  | PVD | |  |  |
| CLD, Osteoporosis | | + | 745(57.44) | 552(42.56) | 10.28  (9.18,11.50) | <0.001 | HT, HD, TCI CBD | | + | 747(62.46) | 449(37.54) | 5.87  (5.21,6.61) | <0.001 |
|  |  | - | 7269(11.61) | 55348(88.39) |  |  |  |  | - | 14539(22.09) | 51292(77.91) |  |  |
|  | |  | MT | |  |  |  | |  | PVD | |  |  |
| CKD, Osteoporosis | | + | 942(57.19) | 705(42.81) | 10.43  (9.43,11.53) | <0.001 | HD, TCI, CBD | | + | 873(61.91) | 537(38.09) | 5.78  (5.18,6.44) | <0.001 |
|  |  | - | 7072(11.36) | 55195(88.64) |  |  |  |  | - | 14413(21.97) | 51204(78.03) |  |  |
|  | |  | MT | |  |  |  | |  | CBD | |  |  |
| Osteoporosis | | + | 3246(55.26) | 2628(44.74) | 13.80  (13.01,14.64) | <0.001 | HT, PVD, DV | | + | 717(76.68) | 218(23.32) | 8.39  (7.21,9.78) | <0.001 |
|  |  | - | 4768(8.22) | 53272(91.78) |  |  |  |  | - | 18606(28.15) | 47486(71.85) |  |  |
|  | |  | PVD | |  |  |  | |  | LMD | |  |  |
| HT, LMD, CLD, CBD | | + | 643(61.18) | 408(38.82) | 5.37  (4.74,6.09) | <0.001 | DM, Gout, CBD | | + | 759(58.29) | 543(41.71) | 5.15  (4.60,5.76) | <0.001 |
|  |  | - | 14262(22.69) | 48601(77.31) |  |  |  |  | - | 14035(21.35) | 51690(78.65) |  |  |
|  | |  | PVD | |  |  |  | |  | LMD | |  |  |
| HD, TCI, CBD | | + | 674(61.05) | 430(38.95) | 5.35  (4.73,6.05) | <0.001 | HT, DM, Gout, CBD | | + | 689(58.09) | 497(41.91) | 5.09  (4.52,5.72) | <0.001 |
|  |  | - | 14231(22.66) | 48579(77.34) |  |  |  |  | - | 14105(21.42) | 51736(78.58) |  |  |
|  | |  | PVD | |  |  |  | |  | PVD | |  |  |
| HT, HD, TCI | | + | 707(60.69) | 458(39.31) | 5.28  (4.69,5.95) | <0.001 | HT, CLD, HD, CBD | | + | 1005(59.89) | 673(40.11) | 5.34  (4.84,5.90) | <0.001 |
|  |  | - | 14198(22.63) | 48551(77.37) |  |  |  |  | - | 14281(21.85) | 51068(78.15) |  |  |
|  | |  | PVD | |  |  |  | |  | CBD | |  |  |
| HT, CLD, HD, CBD | | + | 962(60.16) | 637(39.84) | 5.24  (4.73,5.80) | <0.001 | HT, PVD, HD, TCI | | + | 747(75.68) | 240(24.32) | 7.95  (6.87,9.21) | <0.001 |
|  |  | - | 13943(22.38) | 48372(77.62) |  |  |  |  | - | 18576(28.13) | 47464(71.87) |  |  |
|  | |  | PVD | |  |  |  | |  | PVD | |  |  |
| LMD, CLD, CBD | | + | 764(59.55) | 519(40.45) | 5.05  (4.51,5.63) | <0.001 | HD, CG, CBD | | + | 692(59.76) | 466(40.24) | 5.22  (4.63,5.88) | <0.001 |
|  |  | - | 14141(22.58) | 48490(77.42) |  |  |  |  | - | 14594(22.16) | 51275(77.84) |  |  |

**Note：**HT: hypertension, DM: diabetes mellitus, LMD: lipoprotein metabolism disorder, CG: chronic gastritis, CBD: cerebrovascular disease , CKD: chronic kidney disease , PVD: peripheral vascular disease, MT: malignant tumor, SC: senile cataract, CLD: chronic liver disease, HD: heart disease, TCI: transient cerebral ischemia , DV: dizziness/vertigo.
